# Combination of Plasma Electrolytic Processing and Mechanical Polishing for Single-Crystal 4H-SiC

**DOI:** 10.3390/mi12060606

**Published:** 2021-05-23

**Authors:** Gaoling Ma, Shujuan Li, Xu Liu, Xincheng Yin, Zhen Jia, Feilong Liu

**Affiliations:** School of Mechanical and Precision Instrument Engineering, Xi’an University of Technology, Xi’an 710048, China; magaoling@stu.xaut.edu.cn (G.M.); 2190220037@stu.xaut.edu.cn (X.L.); yinxincheng@stu.xaut.edu.cn (X.Y.); 1190210001@stu.xaut.edu.cn (Z.J.); 105071@xaut.edu.cn (F.L.)

**Keywords:** single-crystal 4H-SiC, mechanical polishing, plasma electrolytic processing, ultra-smooth surface

## Abstract

Single-crystal 4H-SiC is a typical third-generation semiconductor power-device material because of its excellent electronic and thermal properties. A novel polishing technique that combines plasma electrolytic processing and mechanical polishing (PEP-MP) was proposed in order to polish single-crystal 4H-SiC surfaces effectively. In the PEP-MP process, the single-crystal 4H-SiC surface is modified into a soft oxide layer, which is mainly made of SiO_2_ and a small amount of silicon oxycarbide by plasma electrolytic processing. Then, the modified oxide layer is easily removed by soft abrasives such as CeO_2_, whose hardness is much lower than that of single-crystal 4H-SiC. Finally a scratch-free and damage-free surface can be obtained. The hardness of the single-crystal 4H-SiC surface is greatly decreased from 2891.03 to 72.61 HV after plasma electrolytic processing. By scanning electron microscopy (SEM) and X-ray Photoelectron Spectroscopy (XPS) observation, the plasma electrolytic processing behaviors of single-crystal 4H-SiC are investigated. The scanning white light interferometer (SWLI) images of 4H-SiC surface processed by PEP-MP for 30 s shows that an ultra-smooth surface is obtained and the surface roughness decreased from Sz 607 nm, Ra 64.5 nm to Sz 60.1 nm, Ra 8.1 nm and the material removal rate (MRR) of PEP-MP is about 21.8 μm/h.

## 1. Introduction

Single-crystal 4H-SiC has many excellent electrical and chemical properties, such as a wide energy band gap, excellent thermal conductivity, high breakdown electric field, and good chemical stability, which is widely considered as a typical third-generation semiconductor material for the fabrication of power devices working with a high voltage, a high frequency, and under high-temperature conditions. The surface roughness of the single-crystal 4H-SiC substrate seriously affects the performance of the power devices; the electric breakdown field intensity and charge to breakdown have been confirmed to increase with decreasing surface roughness, and a decrease in surface roughness was confirmed to increase the transconductance of a metal oxide semiconductor (MOS) transistor [1]. A smooth surface without scratches and subsurface-damaged (SSD) layers is essential for device applications based on single-crystal 4H-SiC. However, single-crystal 4H-SiC is difficult to polish owing to hard and brittle material properties and strong chemical inertness. Conventional mechanical polishing will cause persistent damage with accompanying residual stress in the single-crystal 4H-SiC surface damage zone, and the optimal selection of diamond grit shapes and sizes is a challenge [2].

In recent years, many polishing techniques based on chemical reactions have been developed for the flattening of SiC substrates, such as chemical mechanical polishing (CMP) [3], plasma-assisted polishing (PAP) [4,5], photochemically combined mechanical polishing (PCMP) [6,7,8], thermal oxidation [9,10], and so forth [11], which can obtain atomically flat single-crystal 4H-SiC surfaces. However, problems of low MRR and pollution still exist.

To improve the MRR of single-crystal 4H-SiC without subsurface damage, several electrochemical methods were proposed [12,13,14]. Li et al. [12] developed a two-step electrochemical mechanical polishing (ECMP) process to polish single-crystal 4H-SiC. Hydrogen peroxide (H_2_O_2_) and potassium nitrate (KNO_3_) were used as the electrolytes to modify the SiC surface into an oxide layer while using colloidal silica slurry as the polishing medium to remove the oxide. Deng et al. [14] developed electrochemical mechanical polishing based on Ceria (CeO_2_) slurry to process a diamond-abrasive-polished surface of single-crystal 4H-SiC and then obtained a smooth surface without subsurface damage.

One common characteristic of the above technologies is that the surface of single-crystal 4H-SiC is modified into an oxide layer with a lower hardness compared to the single-crystal 4H-SiC substrate. Then, the oxide layer is mechanically removed by softer abrasive to achieve a smooth SiC surface. Although the damage-free flattening surface can be realized, MRR is kept low. Therefore, the recent research focuses on developing a precision polishing technology for single-crystal 4H-SiC to achieve higher MRR with required surface roughness.

Plasma electrolytic processing attracts great attention for metal surface modification due to its ability to obtain superior surface properties, cost effectiveness, and be environmental friendly [15,16]. Depending on oxide film conductivity and semiconductor properties, plasma electrolytic processing is normally divided into two main trends: plasma electrolytic oxidation (PEO) and plasma electrolytic polishing (PEPo). PEO, also called micro arc oxidation (MAO), is usually used to coat a ceramic layer with high hardness for the valve metals (Al, Mg, Ti, Zr, Nb, Hf, and Ta). However, for non-valve metals, vapor-gaseous envelope formation is typical, and anodic dissolution usually dominates over the oxide formation in PEPo [17,18]. PEPo is a special electrochemical polishing process used in precision polishing of metal parts at present under the condition of high voltage and environmental saline solution, which can make the surface of the metal part smooth and shiny and obtain a better corrosion resistance with higher MRR [19,20,21]. However, it has not been reported that PEPo is used to polish semiconductors.

This paper proposes a novel polishing technology named plasma electrolytic processing and mechanical polishing (PEP-MP) for single-crystal 4H-SiC polishing, in which the single-crystal 4H-SiC surface is modified into a soft oxide layer by plasma electrolytic processing; then, the oxide layer is mechanically removed by soft abrasive efficiently. NaCl solution is used as the electrolyte for plasma electrolytic processing, and CeO_2_ slurry is used as a polishing medium to remove the oxide layer. Since the CeO_2_ abrasive is softer compared with SiC, the polishing process only removes the oxide layer without introducing any scratches and surface damages. Thus, a flat surface without surface defects is expected with the application of PEP-MP. Furthermore, the mechanism of PEP-MP is discussed.

## 2. The Mechanism of Plasma Electrolytic Processing and Mechanical Polishing

**Figure 1:**
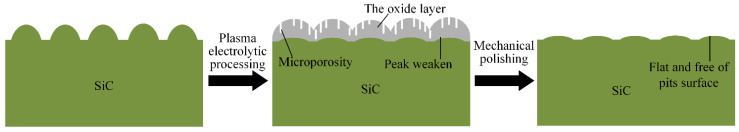
Schematic of plasma electrolytic processing and mechanical polishing (PEP-MP) step.

Figure 1 shows the schematic of PEP-MP step: plasma electrolytic processing of single-crystal 4H-SiC surface and mechanical polishing of the oxide layer. Step 1: the single-crystal 4H-SiC surface is modified into a soft oxide layer, which is mainly made of silicon dioxide by plasma electrolytic processing. Step 2: the soft oxide layer can be mechanically polished by a softer abrasive using the mechanical polishing system. Finally, a flat and pits-free surface can be obtained.

**Figure 2:**
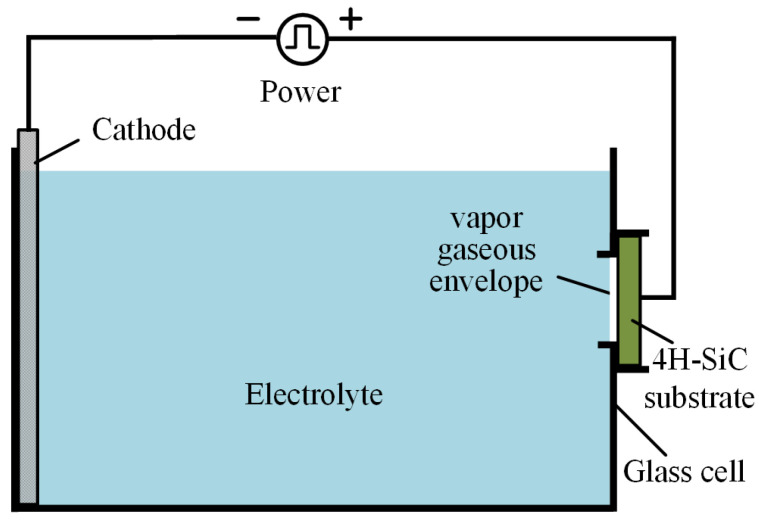
Schematic of plasma electrolytic processing setup.

Figure 2 shows the schematic diagram of the plasma electrolytic processing setup, which consists of a power, electrolyte, cathode, the single-crystal 4H-SiC anode, and an insulated glass electrolytic cell. When the system is charged in a proper current, the instantaneous short circuit of the system results in the evaporation of the electrolyte at the position contact with the workpiece, which generates a vapor gaseous envelope (VGE) composed of water vapor to wrap around the part and isolate the part from the electrolyte [22,23,24]. Under the effect of high electric field, the electrons at the interface between the electrolyte and VGE are instantly accelerated to obtain enough speed and energy rushing to the surface of the part. Then, the electrons directly collide with the water vapor molecules in the VGE and release other electrons. These electrons strike other water vapor molecules, forming electron avalanches. After that, the flow of electrons and ions form a conductive channel that connects the surface of the part to the electrolyte. The channel is filled with high-temperature plasma, which is called the plasma–gas layer [25,26]. This process also brings about complex physicochemical effects, including the generation of ultraviolet light, shockwave, and ultrasonic cavitation. Active substances such as hydroxyl radical (·OH), oxygen radical (·O), hydroperoxide radical (·HO_2_), H_2_O_2_, and O_3_ are produced by chemical effects [27,28]. Through the discharge channel, ·OH, ·O, and ·HO_2_ active substances bombard the surface of the part and react to form an oxide film. The electrons move in the opposite direction at high speed in the discharge channel and collide with the positive ions, leading to the bubble explosion. The plasma–gas layer has a different thickness on the peaks and in the cavities of the part surface, leading to a higher current density on the peaks. A higher oxidation rate can be obtained on the peaks of higher current density, and consequently, higher MRR is for mechanical polishing after plasma electrolytic processing which leads to a smoothing effect rapidly [24,25].

**Figure 3:**
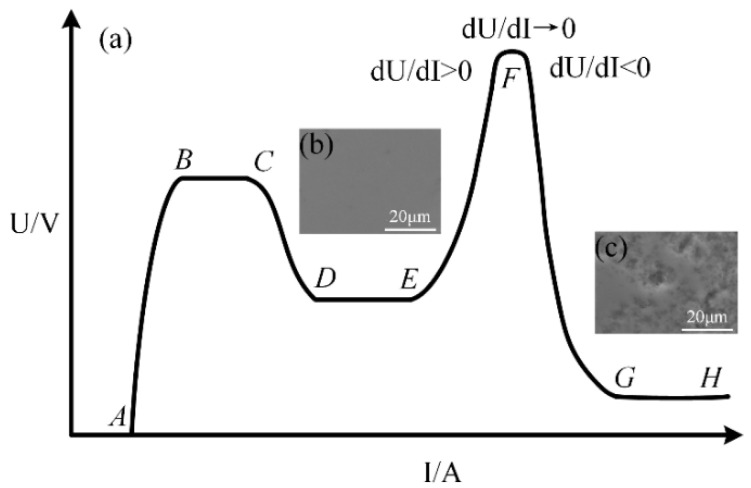
(**a**) I-V curve of gas discharge [29]: (AB) non-self-sustaining discharge region, (BC) Townsend discharge, (CD) subnormal glow discharge, (DE) normal glow discharge, (EF) abnormal glow discharge, (FG) transition to arc, (GH) arc discharge; (**b**) and (**c**) SEM morphologies of single-crystal 4H-SiC after removing the oxide layer formed in regions (EF) and (FG), respectively.

Owing to the exiting of VGE, the discharge between the anode and the electrolyte is gas discharge. Figure 3a shows the classical current–voltage curve of gas discharge [29,30]. Depending on the current, three main types of discharges can be distinguished: (BC) Townsend discharge, (DE) glow discharge, and (GH) arc discharge. In addition, there are four transition regions: (AB) non-self-sustaining discharge region, (CD) subnormal glow discharge, (EF) abnormal glow discharge, and (FG) transition to arc. The gas ionization rate in the glow discharge region (DF) is too low, and the plasma generation rate is relatively low, while the gas ionization rate in the arc discharge region (FH) is too high, and the plasma generation rate is relatively high. According to our experiments, an ultra-smooth surface of single-crystal 4H-SiC shown in Figure 3b can be obtained after removing the oxide layer formed in region (EF) but with low MRR, while high MRR can be obtained with a poor surface full of pits caused by arc discharge shown in Figure 3c. They are all not suitable for surface softening of a semiconductor, which plays a key role in the entire polishing process. In the (EF) abnormal glow discharge region, dU/dI > 0, while dU/dI < 0 in the (FG) transition to the arc region. Therefore, there must be one region around F where dU/dI → 0. The gas ionization rate and plasma generation rate are just suitable for semiconductor surface softening in this region. We call this region the micro arc plasma discharge region, which is different from glow discharge [31] and arc discharge [32].

The oxidation potential of hydroxyl radical is 2.8 eV, which means a strong oxidation property, but it is very unstable. While some hydroxyl radical reacts into water and oxygen, some react with single-crystal 4H-SiC to form silicon dioxide. The electrochemical reactions occurring on the cathode and anode (single-crystal 4H-SiC) can be summarized as follows [8,33,34,35,36].

At the cathode:

2H^+^ + 2e = H_2_, (1)

At the anode (single-crystal 4H-SiC):

OH^−^ − e = •OH, (2)

2•OH + 2•OH = 2H_2_O + O_2_, (3)

SiC+ 4•OH + O_2_ = SiO_2_ + CO_2_+ H_2_O. (4)

During the process of plasma electrolytic processing, with the increase of the oxide layer’s thickness, the upper oxide layer gradually becomes porous and microporosity appears in the oxide layer, as shown in Figure 1. Since the porous oxide layer has a high ion permeability, the active ions can easily reach the SiC surface through the oxide layer [36].

Along the scratched areas and around pits areas, a large amount of covalent bonds formed by carbon atoms and neighboring silicon atoms are broken, which leads to the creation of a large number of dangling bonds, free electrons, and holes [34]. As a result, the scratched areas and pits areas have a high density of available charge carriers, which improves the conductivity of these areas to a large extent, thus leading to the preferential oxidization of these areas. Due to the thinner plasma-gas thickness on the convex peaks, a higher oxidation rate can be obtained. After convex areas, scratch area and pits area are fully oxidized, the rest of the areas begin to be oxidized.

**Figure 4:**
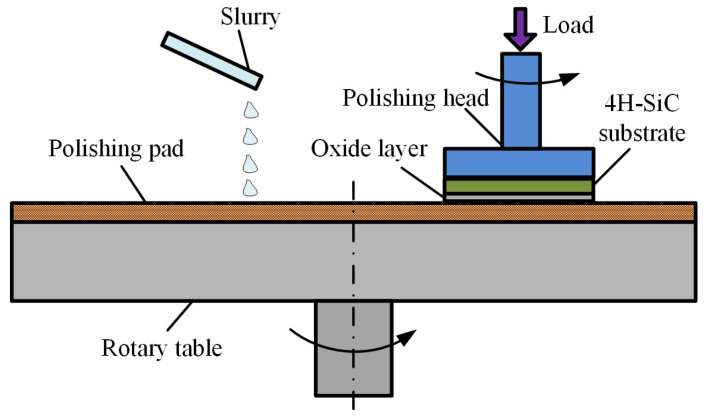
Schematic of mechanical polishing.

The hardness of the oxide layer is much lower than that of single-crystal 4H-SiC substrate, so the oxide layer can be easily removed by a soft abrasive in the mechanical polishing, as shown in Figure 4. The hardness of the abrasive must be lower than SiC so that no scratches or subsurface damage is introduced, and the selected abrasive must be able to remove the oxide layer efficiently. Thus, CeO_2_/SiO_2_ is the optimal choice to polish the oxidized surface due to their ability to remove the oxide efficiently without introducing any damage [10,14,37]. Only the soft oxide layer is removed by the soft abrasive, so the roughness of the oxide/SiC interface determines Ra.

## 3. Experiment of Plasma Electrolytic Processing and Mechanical Polishing

Figure 2 shows the experiment setup employed in plasma electrolytic processing. A unipolar rectangle wave pulse power (frequency from 0.1 to 100 KHz) is applied between the Al cathode and single-crystal 4H-SiC substrate, which was fixed on the anode by a conductive holder. BN-abrasive-lapped single-crystal 4H-SiC substrates (on-axis ±0.2°, off-axis 4 ± 0.2°, N-type) with a thickness of 393 μm and a resistivity of 0.015–0.025 Ω·cm, supplied by TankeBlue Semiconductor Co., Ltd. (Beijing, China) are used. All experiments are conducted on the Si (0001) face, which is commonly used for applications. Sodium chloride (NaCl) aqueous solution with a concentration of 1 wt % is used as the electrolyte. In the mechanical polishing system, as shown in Figure 4, CeO_2_ abrasive particles with the average diameter of 10 nm supplied by Hangzhou ZhiTai Purification Technology Co., Ltd. (Hangzhou, China), and a polyurethane polishing pad with φ230 mm are used. Other experiment parameters are summarized in Table 1.

The substrate is prepared by chemical cleaning before the experiment. First, the substrate is dipped in the sulfuric acid (H_2_SO_4_) (97 wt %) for 10 min to remove contaminants. Second, the substrate is dipped in a concentrated hydrofluoric acid (HF) solution (40 wt %) for 10 min to remove native oxides. Finally, it is rinsed with deionized water for 10 min and blow-dried with pure nitrogen (N_2_) gas.

The surface hardness of single-crystal 4H-SiC after chemical cleaning and after plasma electrolytic processing is measured using a Micro Vickers hardness tester (Mitutoyo HM-200, Mitutoyo, Kanagawa, Japan) to determine the hardness change. The surface morphology of single-crystal 4H-SiC substrate is measured by scanning electron microscopy (SEM) (TESCAN VEGA3, TESCAN ORSAY HOLDING, a.s., Brno, Czech Republic) to investigate the removal of surface defects. Meanwhile, the substrate is observed by X-ray Photoelectron Spectroscopy (XPS) (AXISULTRA, Kratos, Manchester, UK) to confirm its material composition. Thin specimens for cross-sectional microscope observation were prepared and observed using a focused ion beam (FIB) system (JIB-4000, JEOL, Tokyo, Japan). The surface roughness could be measured by scanning white light interferometer (SWLI) (Leica DCM 3D, Leica Microsystems, Wetzlar, Germany), and MRR also can be calculated through the result measured by SWLI.

## 4. Results and Discussion

**Figure 5:**
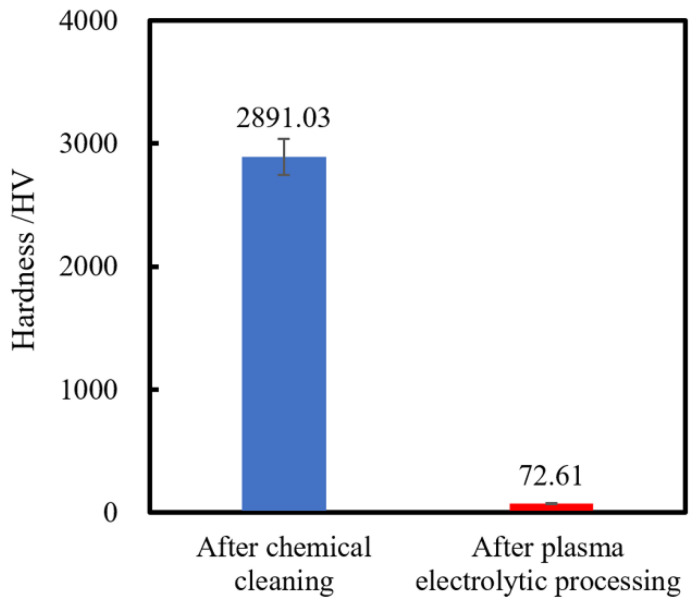
Hardness of SiC measured by Micro Vickers hardness tester.

A Micro Vickers hardness tester with a diamond Vickers indenter is conducted to measure the single-crystal 4H-SiC surface hardness after chemical cleaning and after plasma electrolytic processing, which is shown in Figure 5. In Micro Vickers hardness tests, the maximum load is 0.01 N, and 10 points are measured randomly for each specimen. Figure 5 shows that the hardness of the SiC surface is greatly decreased from 2891.03 to 72.61 HV after plasma electrolytic processing. This result indicates that plasma electrolytic processing is a very effective method for softening the single-crystal 4H-SiC surface. Since the surface is much softer after plasma electrolytic processing, it can be easily mechanically removed by some soft abrasives, such as CeO_2_/SiO_2_.

**Figure 6:**
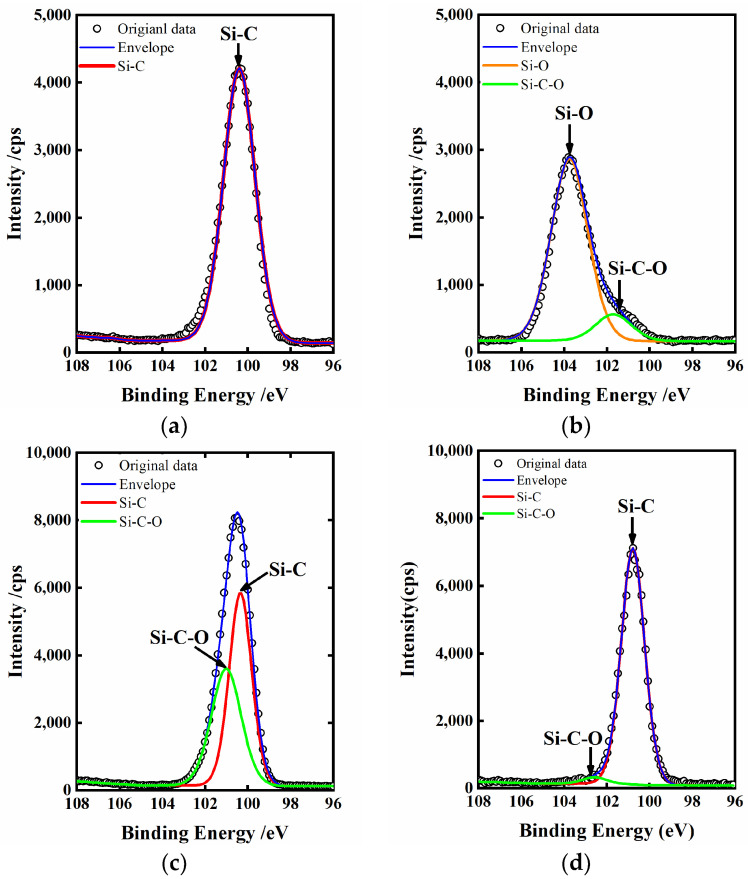
X-ray photoelectron spectroscopy (XPS) analysis of single-crystal 4H-SiC surface: (**a**) Chemical cleaned surface; (**b**) After plasma electrolytic processing for 30 s; (**c**) After HF etching for 10 min; (**d**) After mechanical polishing for 5 min.

Chemical bonding states of the single-crystal 4H-SiC surface are investigated by XPS, and the spectra are obtained with monochromatic Al Ka (1486.7l eV) line at a power of 100 W (10 mA, 10 kV) with the vacuum about 10^−8^ Torr. The charge neutralizer is used to compensate for surface charge effects, and binding energies are calibrated using the C1s hydrocarbon peak at 284.8 eV. Figure 6 shows the Si2p spectra of the single-crystal 4H-SiC surface. A strong peak corresponding to the Si-O bond and a weak peak corresponding to the Si-C-O bond are observed after plasma electrolytic processing for 30 s, as shown in Figure 6b, while there is only the Si-C bond in the chemical cleaned surface shown in Figure 6a. As the thickness of the oxide layer (including SiO_2_ and silicon oxycarbide) exceeds the photoelectron escape depth (<10 nm) in XPS measurements, the Si-C bond is not observed in Figure 6b [38]. This indicates that the chemical cleaned single-crystal 4H-SiC substrate surface is oxidized and forms a soft layer, which is mainly made of SiO_2_ and a small amount of silicon oxycarbide. In order to verify the location of SiO_2_ and silicon oxycarbide, we dip the specimen in Figure 6b in HF for 10 min, and Figure 6c shows the Si2p spectra of the surface after that. The Si-O bond disappears, while the Si-C-O bond is still observed. It indicated that the SiO_2_ layer could be removed by HF etching but not the silicon oxycarbide layer. Comparing Figure 6b,c, it also can be concluded that the silicon oxycarbide layer lies between the SiO_2_ layer and the SiC substrate. The specimen in Figure 6c continued to be mechanical polished by the CeO_2_ slurry for 5 min, and the Si2p spectra of the surface is shown in Figure 6d. The Si-C-O bond with low intensity nearly disappears, and a strong peak corresponding to the Si-C bond can be observed. This result indicates that the silicon oxycarbide layer is almost completely removed by the CeO_2_ slurry.

**Figure 7:**
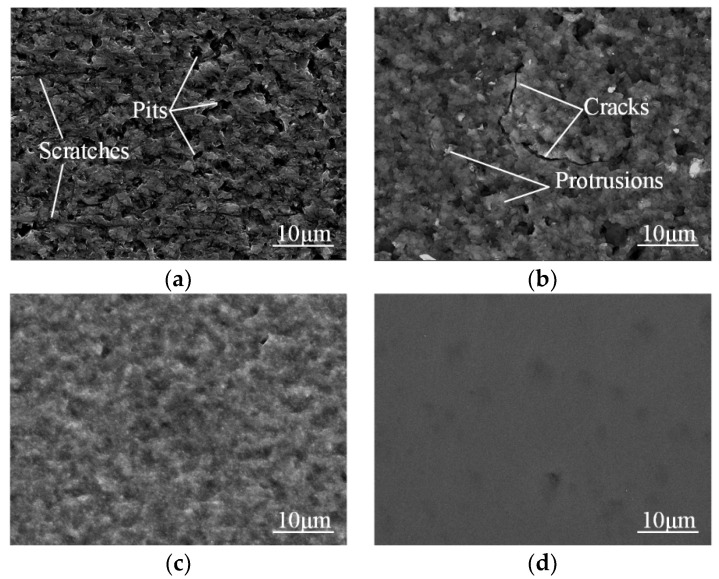
The scanning electron microscopy (SEM) images of single-crystal 4H-SiC surface: (**a**) Chemical cleaned surface; (**b**) After plasma electrolytic processing for 30 s; (**c**) After HF etching for 10 min; (**d**) After mechanical polishing for 5 min.

Figure 7 shows the SEM images of single-crystal 4H-SiC surface by TESCAN VEGA3. Figure 7a shows the chemical cleaned surface, and a lot of deep scratches and pits on the surface can be observed due to the previous slicing and lapping. After processed for 30 s by plasma electrolytic processing, the single-crystal 4H-SiC surface is modified into an oxide layer, as shown in Figure 7b. The protrusions are generated at the defects areas, which indicates that scratches and pits are preferentially oxidized due to the high density of available charge carriers, improving the conductivity of these areas to a large extent [34]. Since the density of SiO_2_ (2.23 g/cm^3^) is lower than that of SiC (3.22 g/cm^3^), the molar mass of SiO_2_ (60 g/mol) is greater than that of SiC (40 g/mol), and the number of Si atoms remains unchanged when SiC is oxidized to SiO_2_; thus, the volume of the oxide expands during the plasma electrolytic processing [9,35]. It is the reason why the scratches are barely visible and the pits seem to deepen after plasma electrolytic processing. The protrusions are generated by the extrusion force, and cracks are formed by the expansion force produced by the subsurface oxidation [35]. Figure 7c shows the SEM image of the surface after the specimen in Figure 6b dipping in HF for 10 min. Almost all of protrusions and cracks disappear owing to the removal of the SiO_2_ layer by HF etching, which is consistent with Figure 6c. Figure 7d shows an SEM image of the PEP-MP-processed area. An ultra-smooth and defect-free surface is obtained.

**Figure 8:**
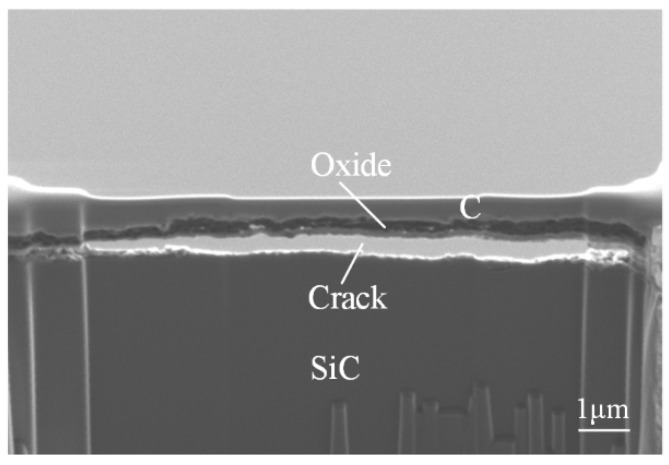
The microscopic view of the cross-sectional layer by focused ion beam (FIB) system.

Figure 8 shows the microscopic view of the cross-sectional oxide layer by the FIB system. The middle layer is the oxide layer, which is covered by a carbon layer for protection in the FIB sample fabrication process. During the FIB sample fabrication process, as the C layer thickness is reduced to a certain extent, some of the oxide layer is peeled off from the SiC substrate, and a crack appears, owing to the stress release between the C layer and the oxide layer, while some of the oxide layer still remain on the SiC substrate. As shown in this image, a poriferous and stratified structure oxide layer is generated, which can be easily removed.

**Figure 9:**
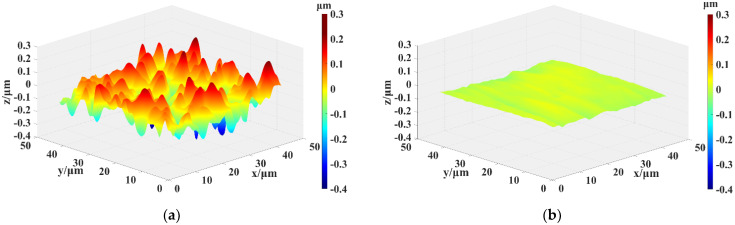
Three-dimensional (3D) morphology images of single-crystal 4H-SiC surface by scanning white light interferometer (SWLI): (**a**) Chemical cleaned surface (Sz 607 nm, Ra 64.5 nm); (**b**) After PEP-MP processed surface (Sz 60.1 nm, Ra 8.1 nm).

Figure 9 shows 3D morphology images of the SiC surface measured by SWLI: (a) as-received surface, (b) after PEP-MP processed surface (plasma electrolytic processing for 30 s and mechanical polishing for 5 min). As shown in Figure 9a, there are many deep scratches, pits, and convex peaks on the chemical cleaned surface, which also can be observed in Figure 7a. Thus, the surface roughness is poor (Sz (p-v) of 607 nm and Ra of 64.5 nm). In Figure 9b, the deep scratches, pits, and convex peaks have almost disappeared, and the surface roughness dramatically decreases to Sz of 60.1 nm and Ra of 8.1 nm. The plasma–gas thickness on the peaks is thinner than that on other areas, leading to ·OH reaching the peaks preferentially, and consequently, a higher oxidation rate. The scratched areas and pits areas have a high density of available charge carriers [34], which improves the conductivity to a large extent, thus leading to the preferential oxidization of these areas.

**Figure 10:**
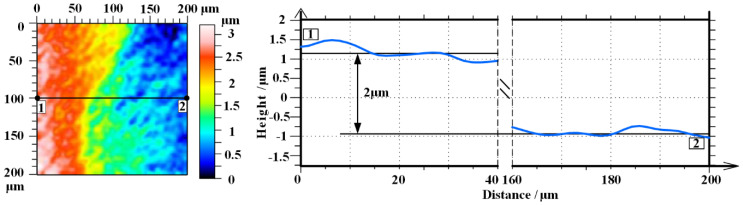
Oxidation depth of the chemical cleaned surface after PEP-MP for 30 s (l chemical cleaned area, 2 polished area).

Figure 10 shows the oxidation depth after removing the oxide layer by a scanning white light interferometer. Area 1 is chemical cleaned area, while area 2 is a polished area. The oxidation depth is about 2 μm according to the cross-sectional view along 1−2. Therefore, we can calculate that the material removal rate (MRR) of PEP-MP is about 21.8 μm/h, while the maximum MRR of other single-crystal 4H-SiC polishing technology is about 23 μm/h in Yang’s [39] research, who use ECMP for the sliced single-crystal 4H-SiC planarization. The MRR of PEP-MP for BN-abrasive-lapped single-crystal 4H-SiC is close to the maximum MRR of ECMP for the sliced single-crystal 4H-SiC, but it is much bigger than the maximum MRR (3.62μm/h) of ECMP for the diamond-abrasive-lapped single-crystal 4H-SiC [14]. Taking the density and molar mass of SiC and SiO_2_ into consideration, a thickness of about 2 μm of 4H-SiC oxidized and it indicates that the SiO_2_ layer thikness *h* is at least 4.2 μm. According to the Micro Vickers hardness test results, the indentation depth *d* of about 0.4 μm can be calculated. *h*/*d* = 0.4/4.2 < 0.1, and this verifies the hardness testing results are reliable without consideration of the substrate effect [40]. These results all demonstrate that PEP-MP is an effective technology for flattening SiC.

## 5. Conclusions

To realize high-efficiency polishing for single-crystal 4H-SiC, the PEP-MP method combines plasma electrolytic processing, and soft abrasive polishing is proposed. Experiments were conducted, and the results show that the method is feasible. The conclusions are summarized as follows:

(1) In the process of plasma electrolytic processing, the single-crystal 4H-SiC surface is oxidized to soften the oxide layer, which is mainly made of SiO_2_ and a small amount of silicon oxycarbide with a very high oxidation rate. The silicon oxycarbide is located between the SiO_2_ layer and the SiC substrate.

(2) It is confirmed that the surface hardness of SiC was greatly decreased by plasma electrolytic processing, which made the oxide layer can be easily removed using softer abrasive such as CeO_2_.

(3) Preferential oxidization happens in the scratched and pits areas those with high conductivity. The plasma-gas thickness on the peaks is thinner than that on other areas, leading to a higher oxidation rate on the peaks.

(4) Applying PEP-MP to a BN-abrasive-lapped SiC surface, an ultra-smooth and defect-free surface is obtained. Meanwhile, the surface roughness decreases from Sz 607 nm, Ra 64.5 nm to Sz 60.1 nm, Ra 8.1 nm. The material removal rate (MRR) of PEP-MP is about 21.8 μm/h through the observation by SWLI.

## Figures and Tables

**Table 1:** Experiment parameters.

Parameters	Value
Voltage	200 V
Frequency	20 kHz
Duty cycle	50%
Electrolyte	DI Water + NaCl 1 wt %
Electrolyte Temperature	25 °C
Slurry	DI Water + CeO_2_ 30%
Load	74 kPa
Polishing head rotation speed	100 rpm
Polishing pad rotation speed	1400 rpm
Plasma electrolytic processing time	30 s
Mechanical polishing time	5 min
